# Immunoexpression of HSP27 does not seem to influence the prognosis of oral tongue squamous cell carcinoma

**DOI:** 10.1590/0103-6440202305036

**Published:** 2023-12-22

**Authors:** Ondina Karla Mousinho Rocha Torres, Luiz Arthur Barbosa da Silva, Roseana de Almeida Freitas, Éricka Janine Dantas da Silveira, Márcia Cristina da Costa Miguel

**Affiliations:** 1 Post graduate Program in Dental Sciences, Department of Oral Pathology, Federal University of Rio Grande do Norte, Natal, Rio Grande do Norte, Brazil; 2 Department of Oral Pathology, Federal University of Rio Grande do Norte, Natal, Rio Grande do Norte, Brazil

**Keywords:** squamous cell carcinoma, tongue, immunohistochemistry, heat-shock proteins, prognosis

## Abstract

New methods of early detection and risk assessment have been studied aiming to predict the prognosis of patients and directing a specialized treatment of the oral tongue squamous cell carcinoma (OTSCC). In this context, several molecular biomarkers have been investigated for this purpose, and, among them, the heat shock protein 27 (HSP27) can be named. The study aimed to analyze whether heat shock protein 27 (HSP27) exerts any influence on OTSCC, correlating its immunoexpression with clinicopathological parameters, and patient survival. The sample comprised 55 OTSCC cases and 20 normal oral mucosa specimens. The malignancy grading systems proposed by the WHO in 2005, Brandwein-Gensler et al., and Almangush et al. were applied in a histomorphological study. HSP27 expressions were evaluated through the Immunoreactivity Score System (IRS). Significant values were considered at p <0.05 for all statistical tests. Higher IRS results were observed for normal oral mucosa specimens when compared to OTSCC cases (p <0.001). No significant associations between HSP27 immunostaining, the analyzed clinicopathological parameters and patient survival were observed. The results of the present study indicate lower HSP27 expression in OTSCC cases compared to normal oral mucosa specimens. Thus, HSP27 expression does not seem to influence patient prognosis.

## Resumo

Novos métodos de detecção precoce e avaliação de risco estão sendo estudados com o intuito de predizer o prognóstico dos pacientes e direcionar um tratamento diferenciado. Neste contexto, vários biomarcadores moleculares têm sido investigados com esta finalidade, dentre eles a heat shock protein 27 (HSP27). Esta pesquisa objetivou analisar se a HSP27 exerce alguma influência nos carcinomas de células escamosas de língua oral (CCELO), correlacionando a sua imunoexpressão com parâmetros clinicopatológicos e com a sobrevida dos pacientes. A amostra foi constituída por 55 casos de CCELO e 20 espécimes de mucosa oral normal. Os sistemas de gradação de malignidade propostos pela OMS em 2005, Brandwein-Gensler et al. e Almangush et al. foram aplicados em um estudo histomorfológico. A expressão da HSP27 foi avaliada através do Sistema de Escore de Imunorreatividade (IRS). Para todos os testes estatísticos foram considerados valores significativos com p<0,05. Foi observado um maior IRS para a mucosa oral normal quando comparado aos casos de CCELO (p<0,001). Não foram encontradas associações significativas entre a imunomarcação da HSP27 com os parâmetros clinicopatológicos analisados e com a sobrevida dos pacientes. Os resultados do presente estudo indicam uma menor expressão da HSP27 nos casos de CCELO quando comparados aos espécimes de mucosa oral normal. Assim, a expressão da HSP27 parece não influenciar o prognóstico dos pacientes.

## Introduction

Oral cancer is the 11th most frequent cancers worldwide. In this scenario, squamous cell carcinoma (SCC) is the most prevalent, accounting for over 90% of all oral cavity cancers ^1^. Despite the enormous progress in recent decades, mortality rates are high and related to late diagnoses, the aggressive course of the disease, resistance to treatment and recurrence ^2^.

Molecular markers may play a significant role in detecting prognoses, assessing disease progression and choosing appropriate treatment. Heat shock proteins (HSPs), highly conserved molecular chaperones, are noteworthy among several biomarkers explored in human cancers. These proteins regulate protein synthesis, folding, assembly and degradation. In addition to their cytoprotective effects, studies report that HSPs can induce malignant cell proliferation, angiogenesis and resistance to treatment ^3^, ^4^.

Several HSP isoforms have been reported, and HSP27 has recently received increased attention due to its association with tumor prognosis and resistance to therapeutic strategies in various types of cancer ^5^. The role of HSP27 in oral squamous cell carcinomas is not well understood. In this context, the aim of the present study was to analyze whether HSP27 exerts any influence on oral tongue squamous cell carcinomas (OTSCC), correlating its immunoexpression with clinicopathological parameters and patient survival.

## Methodology

A total of 55 OTSCC cases and 20 normal oral mucosa specimens from patients submitted to surgical treatment to remove benign lesions, whose borders displayed non-neoplastic oral epithelia, were assessed in the present study. The research was submitted and approved by the Research Ethics Committee (protocol nº 2.155.630).

All OTSCC clinical information was retrieved from medical records. Only cases located in the anterior two thirds of the tongue with minimum follow-up information at 60 months were included in the survival analysis. The investigated outcomes comprised global survival, disease-specific survival, and disease-free survival. Cases submitted to chemotherapy and/or radiotherapy prior to surgical treatment were excluded.

### Morphological analysis

Hematoxylin and eosin-stained slides were scanned using a Pannoramic MIDI Scanner (3DHISTECH, Budapest, Hungary, H-1121) and visualized through the Pannoramic Viewer software 1.15.2 (3DHISTECH, Budapest, Hungary, H-1121). OTSCC samples were assessed and classified according to the grading system proposed by the WHO (6), the histological risk malignancy model developed by Brandwein-Gensler *et al.* ^7^, and the BD model, developed by Almangush *et al*. ^8^.

### Immunohistochemistry analysis

Histological sections (3 μm) of each sample were obtained from paraffin-embedded specimens. The sections were dewaxed, rehydrated, and subjected to antigen recovery with Trilogy (Cell Marque, CA, USA). Subsequently, endogenous peroxidase blocking, washing under running water, and incubation in protein blocker (ThermoScientific, Runcorn, UK) were performed, followed by two washes with tris-hydroxymethylaminomethane (TRIS, Sigma Chemical, St. Louis, MO, USA) (pH 7.4). The slides were then subjected to anti-HSP27 (Clone G31; Cell Signaling Technology, 1:4000 dilution; incubation 60 min; antibody validation by manufacturer´s system control strategies) and incubation using the Hidef system (Cell Marque, CA, USA) for 60 min. After incubation, development with diaminobenzidine (DAB, Sigma Chemical, St. Louis, MO, USA), counterstaining with Mayer's hematoxylin, and slide assembly with Permount resin (Fisher Scientific Inc., Fair Lawn, NJ, USA) were performed. Negative controls were obtained by replacing the primary antibody with 1% bovine serum albumin (BSA - Bovine serum albumin) in a buffer solution. A breast carcinoma specimen was used as a positive control.

HSP27 immunoexpression detection, performed by a single, previously trained examiner during two different moments, was based on cytoplasmic labeling, considered positive when cells presented brownish pigments. All specimens were scanned using the Pannoramic MIDI Scanner and the images were visualized through the Pannoramic Viewer software 1.15.2. Five fields were selected for all cases, at a 33.33 amplitude, corresponding to a 400x light microscope magnification. HSP27 immunoexpression detection was carried out on the tumor front in OTSCC tumor samples and in the basal, parabasal and upper layers in normal oral mucosa samples. The methodology proposed by Luz *et al.* ^9^ was applied, with adaptations, using the Immunoreactivity Scoring System (IRS) and assessing the protein based on cytoplasmic immunostaining intensity and the percentage of immunopositive tumor cells in five selected fields. The following scores were considered regarding marking intensity: 0 (negative), 1 (weak), 2 (moderate) and 3 (strong). The percentage of immunolabelled cells was classified as 0 (negative), 1 (1-10%), 2 (11-50%), 3 (51-80%) and 4 (> 80%). Subsequently, the multiplication of the score of the labeling intensity was performed using the percentage score of immunolabelled cells, resulting in the IRS, ranging from 0 -12. The total IRS of each case was established according to the IRS median of the five analyzed fields.

### Statistical analyses

The Mann-Whitney and Kruskal-Wallis tests were used to verify significant differences between groups, and Pearson's Chi-square test and Fisher's exact test were used to search for significant associations, using the SPSS software, version 22 (SPSS Inc.). The survival analysis was performed using the Stata/IC software, version 12.0 (StataCorp, College Station, TX), by applying the Kaplan Meier method and the log-rank test (to obtain the statistical significance of the differences between the obtained survival curves). Five-year global survival curves for each of the variables were constructed. A Cox regression (Cox proportional hazards model) was used to determine the hazard ratio (HR) and its 95% confidence interval. Statistical significance was set at p <0.05.

## Results

The distribution of OTSCC cases according to epidemiological and clinicopathological parameters is detailed in Table 1. Most individuals were male (37/67.3%), with a male to female ratio of 2.05:1. A total of 42 individuals (76.4%) were smokers and 29 (52.7%) were alcoholics. Local recurrence was observed in 13 individuals after treatment, after an average of 12.85 months. Of the 55 patients, 15 (27.3%) died after 13.60 months, all related to the progression of the underlying disease (OTSCC).

**Table 1:** Distribution of OTSCC cases according to epidemiological and clinicopathological parameters

Parameter	n (%)
*Sex*	
Male	37 (67.3)
Female	18 (32.7)
*Age*	
Up to 60 years old	22 (40.0)
≥ 60 years old	33 (60.0)
*Smoking*	
No	4 (7.3)
Yes	42 (76.4)
Not informed	9 (16.3)
*Alcoholics*	
No	17 (31.0)
Yes	29 (52.7)
Not informed	9 (16.3)
*Tumor size (T)*	
T_1_ -T_2_	34 (61.8)
T_3_ -T_4_	17 (30.1)
Not informed	4 (8.1)
*Lymph node involvement (N)*	
N(-)	31 (56.4)
N(+)	20 (36.3)
Not informed	4 (7.3)
*Distant metastasis (M)*	
M(-)	50 (90.9)
M(+)	0 (0.0)
Not informed	5 (9.1)
*Local recurrence*	
Absent	42 (76.4)
Present	13 (23.6)
*Second primary tumor*	
Absent	50 (90.9)
Present	5 (9.1)
*Lymph node metastasis after treatment*	
Absent	50 (90.9)
Present	5 (9.1)
*WHO grading*	
Well differentiated	31 (56.4)
Moderately differentiated	16 (29.1)
Poorly differentiated	8 (14.5)
*Brandwein-Gensler risk*	
Low risk	1 (1.8)
Intermediate Risk	30 (54.5)
High risk	24 (43.6)
*BD model*	
Low risk	3 (5.5)
Intermediate Risk	14 (25.5)
High risk	38 (69.0)

Regarding histological characteristics as classified by the WHO grading frequency (6) and histological risk as proposed by Brandwein-Gensler *et al.* ^7^ and by Almangush *et al.* ^8^ (BD model), most cases were classified as well differentiated (56.4%) according to WHO, intermediate risk (54.5%) according to Brandwein-Gensler *et al.* ^7^ and high risk (69.1%) according to the BD model.

**Figure 1:**
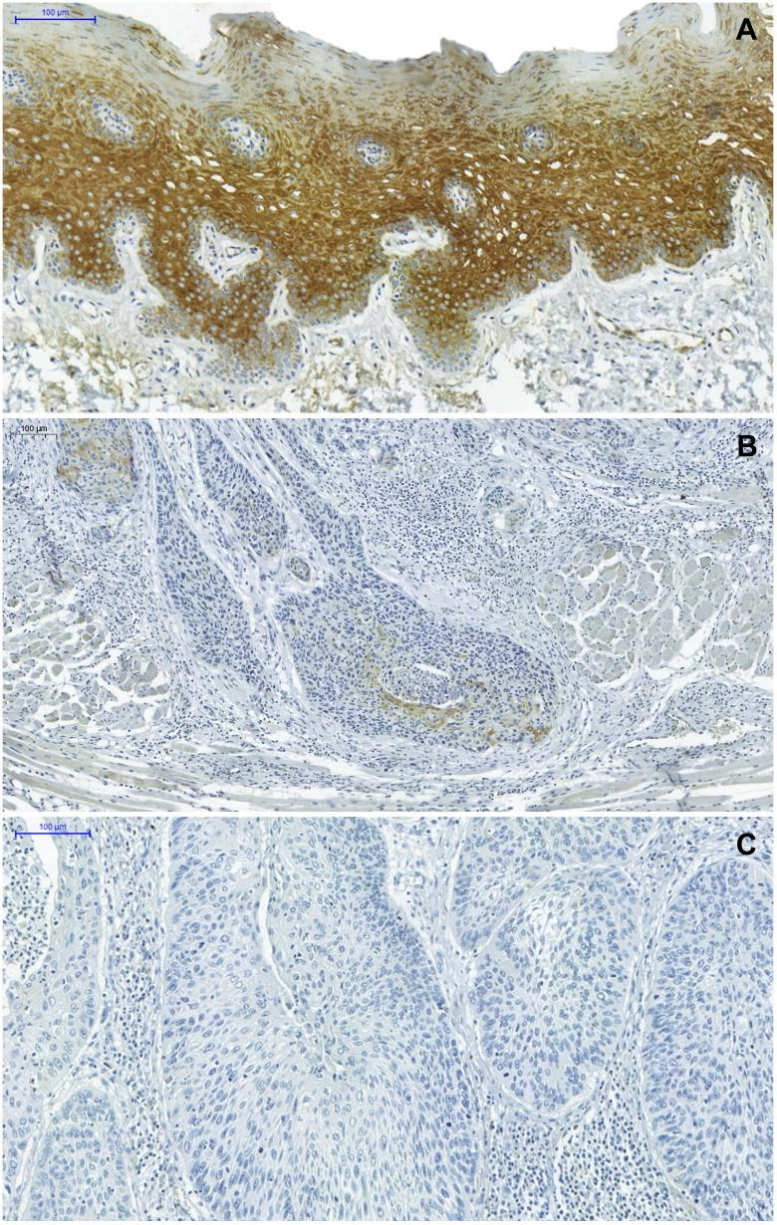
HSP27 immunoexpression in normal oral mucosa and OTSCC cases. a) Higher amounts of immunolabelled cells are present in the parabasal layer and upper layers of normal oral mucosa epithelium, with labeling intensity ranging from moderate to intense; b) A lower number of immunolabelled cells with a marked moderate is detected in the OTSCC invasion front,. c) In some cases, tumor cells were not labelled (Pannoramic Viewer - 100μm, 200μm, 100μm, respectively)

Cytoplasmic HSP27 expression was observed in all OTSCC cases. Immunopositivity distribution in the parabasal layer and upper layer cells was observed with certain frequency in normal oral mucosa specimens, ranging from moderate to intense (Figure 1a). A lower amount of immunolabelled cells was observed in OTSCC cases compared to normal oral mucosa samples, displaying predominantly weak to moderate intensity (Figure 1b and c). A statistically significant difference was noted between OTSCC and normal mucosa samples, with higher IRS detected in the latter (Table 2).

**Table 2:** Comparative analysis of HSP27 immunoexpression in OTSCC and normal oral mucosa according to the Intensity Score System (IRS)

	Group	N	Median	Q25-75	Ranks	*P*
IRS	OTSCC	55	3.0	2.0-4.0	30.34	<0.001*
	Normal oral mucosa	20	8.0	4.5-11.0	59.08	

Q25-75: 25 and 75 Quartiles; Ranks: Avg. Mann-Whitney test. Legend: * Significant result.

HSP27 expression was categorized based on the minimum IRS obtained for the normal oral mucosa samples (4.0), resulting in two groups: lower (IRS up to 4.0) and higher (IRS over 4.0) expression. No significant associations between the groups were observed. However, concerning the WHO classification ^6^, all 8 cases classified as poorly differentiated presented low HSP27 expression (Table 3).

**Table 3:** Association of HSP27 expression in relation to clinicopathological parameters

Parameter	HSP27		*P*
	Up to 4.0n (%)	>4.0n (%)	
Categorized T *			
T1-T2	28 (82,3)	6 (17.7)	1.000
T3-T4	14 (82.3)	3 (17.7)	
Categorized N *			
N(-)	27 (87.0)	4 (13.0)	0.289
N(+)	15 (75.0)	5 (25.0)	
Clinical Staging *			
I-II	20 (86.9)	3 (13.1)	0.487
III-IV	22 (78.5)	6 (21.5)	
*Local recurrence*			
Absent	35 (83.3)	7 (16.7)	0.100
Present	11 (84.6)	2 (15.4)	
*Second primary tumor*			
Absent	42 (84.0)	8(16.0)	1.000
Present	4 (80.0)	1 (20.0)	
*Lymph node metastasis after treatment*			
Absent	43 (86.0)	7 (14.0)	0.184
Present	3 (60.0)	2 (40.0)	
*Distant metastasis after treatment*			
Absent	45 (84.9)	8 (15.1)	0.303
Present	1 (50.0)	1 (50.0)	
*WHO staging*			
Well / Moderate	38 (80.8)	9 (19.2)	0.327
Poorly	8 (100.0)	0 (0.0)	
*Brandwein-Gensler risk*			
Low / Intermediate	25 (80.6)	6 (19.4)	0.716
High	21 (87.5)	3 (12.5)	
*BD model*			
Low / Intermediate	15(88.2)	2(11.8)	0.705
High	31(81.5)	7(18.5)	

Fisher's Exact Test for n <5 and Pearson's Chi-Square for n> 5. Caption: * 4 cases without information.

**Figure 2:**
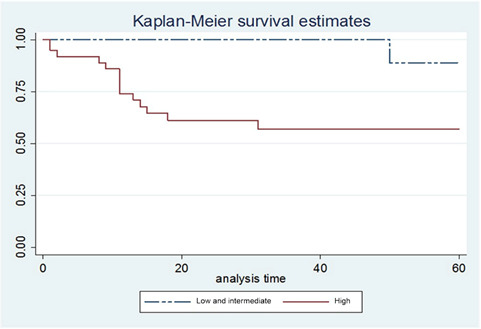
Kaplan-Meier curves with global survival estimates for the BD model

The disease-specific survival time coincided with overall survival time, since all deaths were associated with cancer. Of the 55 OTSCC cases, 40 patients (72.7%) remained alive for 5 years, while 15 (27.3%) died. Regarding death cases (n:15), the mean time to patient death was 13.60 months. No significant association was found between HSP27 expression, overall survival and disease-free survival. On the other hand, a significant association between the BD model, death and overall survival was noted, with the high-risk score associated to patient death and lower survival times (Figure 2) (Table 4).

**Table 4:** Association of deaths overall survival and disease-free survival in relation to histopathological and immunohistochemical parameters

Parameter	Death			*p*
	No n (%)		Yes n (%)	
*WHO staging* Well / Moderate Poorly	32(80.0) 8(20.0)		15(100.0) 0(0.0)	0.091
*Brandwein-Gensler risk* Low / Intermediate High	25 (62.5) 15 (37.5)		6 (40.0) 9 (60.0)	0.134
*BD model* Low / Intermediate High	16 (40.0) 24 (60.0)		1 (6.7) 14 (93.3)	0.022*
*HSP27* Up to 4 Over 4	33 (82.5) 7 (17.5)		13 (86.7) 2 (13.3)	1.000
Parameter	No. of deaths	Overall survival in 5 years (95% CI)	HR (95% CI)	*P*
*WHO staging* Well / Moderate Poorly	15 0	60.37 (42.31-74.37) 100.0	a	0.062
*Brandwein-Gensler risk* Low / Intermediate High	6 9	74.68 (50.93 - 88.13) 57.43 (33.70 - 75.37)	1.93 (0.68 - 5.43)	0.197
*BD model* Low / Intermediate High	1 14	88.89 (43.30 - 98.36) 56.96 (37.94 - 72.12)	7.62 (1.01 - 58.14)	0.019*
*HSP27* Up to 4 Over 4	13 2	65.20 (47.24 - 78.35) 76.19 (33.22 - 93.51)	0.78 (0.17 - 3.49)	0.751
Parameter	No. of events	Five-year disease-free survival (95% CI)	HR (95% CI)	*P*
*WHO staging* Well / Moderate Poorly	25 2	35.98 (20.96-51.23) 71.43 (25.82-91.98)	0.27 (0.06-1.15)	0.054
*Brandwein-Gensler risk* Low / Intermediate High	14 13	42.50 (22.36 -61.35) 38.79 (18.55 -58.72)	1.27 (0.59 - 2.70)	0.526
*BD model* Low / Intermediate High	7 20	41.79 (14.51-67.40) 39.76 (23.08 -55.98)	1.62 (0.68 - 3.85)	0.257
*HSP27* Up to 4 Over 4	22 5	42.05 (25.95-57.32) 33.86 (5.75 -66.35)	1.28 (0.48 - 3.39)	0.612

Caption: CI: confidence interval, HR: odds ratio, * significant result.

## Discussion

Oral cancer arises from multiple molecular events that develop in susceptible individuals. Despite being influenced by extrinsic risk factors, genetic predisposition also plays an important role in oral cancer pathogenesis. In recent years, developments in molecular biology techniques and genetic approaches have opened new dimensions in the understanding of oral cancer, leading the search for molecular biomarkers with predictive value in identifying high-risk patients ^10^.

Regarding histopathological grades, no reproducibility between the systems applied herein and their classifications was found, attesting the findings reported by Strieder *et al*. ^11^ and Dik *et al.* ^12^. The BD model developed by Almangush *et al.* ^(^ ^8^ was the only model indicating correlations with overall patient survival and death. Strieder et al. ^11^ and Silva et al. ^13^ who found positive correlations regarding patient survival with SCC of the lip and OTSCC, respectively, with an association observed between high risk and worse prognosis evidenced similar findings.

Limitations regarding current clinical staging systems and histopathological degrees are still a concern up to the present time. Therefore, pathologists and oncologists remain on the hunt for an ideal indicator that assists in the prognosis and choice of appropriate patient treatment ^13^, ^14^.

It has recently been reported that HSP27, a major molecular chaperone, plays an important role as a prognosis marker and is associated with chemoresistance in various types of cancer. Studies indicate that the high expression of this protein contributes to tumor progression through different mechanisms and that its anti-apoptotic and pro-survival activities play crucial roles in tumorigenesis ^5^.

Malignant cells usually present high HSP27 expression, in order to meet the high metabolic requirements and signal transduction required to maintain cell survival and high HSP27 expression has been correlated with tumor aggressiveness and low patient survival rate ^5^, ^15^. In contrast, some studies have demonstrated that low HSP27 expression is associated with lymph node metastasis and worse prognoses ^16^. Considering this, we suggest that the correlation between protein expression and prognosis varies depending on the type of cancer.

Concerning HSP27 immunoexpression in the present study, a statistically significant value was found regarding the IRS (p <0.001), indicating decreased HSP27 immunostaining in OTSCC cases, in agreement with the study reported by Muzio *et al.* ^17^ who observed over 80% immunopositivity in normal oral mucosa epithelia samples and up to 60% in OTSCC cases.

Although the mechanisms involved in decreased HSP27 expression in oral carcinomas are not addressed properly in the literature, the study reported by Wang *et al.* ^18^ provides evidence that the HSPB1 gene, responsible for the control of HSP27 production, undergoes hypermethylation in the promoter region in OTSCC cases. Thus, this negatively regulated gene would lead to low HSP27 expression in these tumors. Zhang *et al.* ^15^ report that certain post-translational modifications, such as phosphorylation, acetylation and sumoylation, modulate the activation of heat shock factor 1 (HSF1), the main HSP27 transcription regulator in mammals. Karri *et al.* ^3^ observed high HSP27 expression in normal oral mucosa and in well-differentiated OSCC cases, low levels in mild epithelial dysplasia’s. However, a gradual increase in HSP27 expression was observed as the progression of dysplasia occurs. These authors suggest that the low expression of HSP27 can be considered as a molecular indicator of initial dysplastic change and an increase in its expression may indicate a likely transformation into an OSCC. Ramalingam et al. ^19^, highlighted that the low expression of HSP27 found in oral epithelial dysplasia’s may result in a defective cytoprotection against mutagenic environmental factors, enabling the transformation from epithelial dysplasia to OSCC.

Lee *et al.* ^20^ observed a significantly higher HSP27 expression in OTSCC cases associated with the specific areca nut chewing habit when compared to normal oral mucosa specimens. The authors believe that the heavy metals found in areca nuts generate reactive oxygen species during mastication, leading to increased HSP27 expression. This suggests that the deregulation observed in OTSCC cases can be attributed to different etiological carcinogenesis-involved factors. In turn, Iqbal *et al.* ^21^ observed no difference in HSP27 immunopositivity between normal tissues and esophageal SCC, and hypothesized that this outcome may be justified by continuous food stress or pathological conditions. In this sense, since oral cavity is vulnerable to the exposure of several exogenous agents and suffers direct impacts caused by mastication, characterization of a continuous stress in this location may support the high expression of this protein in normal oral mucosa.

No significant associations were found between HSP27 immunoexpression and clinicopathological parameters in the present study. However, higher tendency on low expression of HSP27 was noted in cases classified as poorly differentiated on WHO grading system. Similar findings were evidenced by Muzio *et al*. ^(^ ^17^ and Wang *et al.* ^(^ ^22^, indicating lower HSP27 marking in relation to the WHO histopathological grading, leading to low protein expression in poorly differentiated SCC and OTSCC cases. Some studies have associated HSP27 expression with increased cell differentiation, suggesting that HSP27 may be a keratinocyte differentiation of ^23^, ^24^. This may also justify the high expression of this protein in the parabasal and spiny layers of the normal oral mucosa observed herein.

No significant associations were found between HSP27 immunoexpression and disease-free and overall survival analysis, contrasting with the findings reported by Wang *et al.* ^22^ and Mohtasham *et al*. ^23^, who reported that low HSP27 expression in SCC and OTSCC cases is directly correlated to lower patient survival rates. On the other hand, Sheng *et al*. ^25^ and Han *et al*. ^26^ when assessing lung and colorectal carcinomas, respectively, observed that higher HSP27 expression was associated with lower survival rates. Therefore, the divergences found in the literature on the HSP27 expression, and the prognosis of carcinoma patients may be associated to histological diversity and heterogeneous nature of the investigated tumors.

The present study presents as limitation the limited sample size due to the pre-established inclusion criteria, especially the five years of follow-up necessary in order to obtain data on recurrence and patient survival. Furthermore, the expression of the protein assessed in this research, through immunohistochemistry, allows an excellent in situ analysis, but it does not help to identify whether there are specific molecular alterations, which can conclusively clarify the role of this protein in the OTSCC pathogenesis.

In conclusion, a lower HSP27 expression in OTSCC cases when compared to normal oral mucosa were observed in the present study. No significant associations were observed between HSP27 immunostaining, clinicopathological parameters and patient survival, suggesting that HSP27 expression does not seem to influence OTSCC prognosis.
